# Pyrethroid Insecticides in Households from Urban Areas: An Association of the 3-PBA Metabolite and Hand Wipes

**DOI:** 10.5334/aogh.2746

**Published:** 2020-06-05

**Authors:** Jadsada Kunno, Parichat Ong-artborirak, Pongsaya Panicharoen, Mark Gregory Robson, Wattasit Siriwong

**Affiliations:** 1 College of Public Health Sciences, Chulalongkorn University, Bangkok, TH; 2 Center for Risk Analysis and Health Surveillance, Chulalongkorn University, Bangkok, TH; 3 School of Environmental and Biological Sciences, Rutgers University, New Brunswick, New Jersey, US

## Abstract

**Background:**

Pyrethroid exposure in the household environment affects children directly via inhalation or dermal exposure. Hand wipes can effectively predict pyrethroid exposure to young children along with the children’s activities. The main purpose of this study is to identify the relationship between 3-PBA metabolites, hand wipe sample concentrations and multiple exposure factors, within the population of households with young children in urban Bangkok, Thailand.

**Methods:**

Interviews were conducted with the parents of 80 children (aged 2–3 years). Urine was collected to analyze for 3-PBA metabolites and hand wipe samples were collected to analyze for cypermethrin. Both were analyzed by gas chromatography (GC/MS).

**Results:**

A Spearmen’s correlation test of the increase of 3-PBA metabolites was significantly associated with an increase in hand wipe samples (cypermethrin) in children (r_s_ = 0.226–0.274, *p*-value < 0.05). The binary logistic regression test presented an association between exposure factors with 3-PBA metabolite concentration. Gender presented a significant association with 3-PBA metabolites (*p*-value = 0.035, OR = 0.326, 95% CI 0.115–0.926), and frequency of bare feet inside the household presented a significant association with 3-PBA metabolites (*p*-value < 0.01, OR = 7.072, 95% CI 1.707–29.291). In addition, exposure factors were not significantly associated with wipe sample concentration (cypermethrin) but showed high risk of exposure to young children.

**Conclusions:**

Suggestions to reduce the risk from long-term pyrethroid insecticide exposure to children living in households include increased education, awareness, and management.

## Background

Pyrethroid (PYR) insecticide residues are prevalent throughout the world. However, there is little information available to the general public. Studies have shown that insecticide concentration in homes have little variability over two-month long time frames [1,2]. The use of PYR insecticide in low-income families is common due to poor housing conditions and pest outbreaks. However, pesticide exposure data in low income households is limited. Some evidence has been presented showing that young children aged 1–3 years old may be a high-risk group for household PYR exposure by multiple routes [3]. The role of children’s activities leading to pesticide exposure was assessed by comparing the amount of pesticides in the hands of children with the same activity observed over a four-hour period. Children between 2-5 years of age show child behaviors that are quantifiable to the place where the behavior occurs when compared to picking up pesticides by their hands. The amount of pesticides received at the time of filming can also be correlated with the levels of pesticides on surfaces and toys [19,20,21]. However, children have different activity patterns (e.g., crawling), they are closer to the ground and eat more food and water per kilogram than adults. In addition, children may be at greater risk of exposure to pollution due to higher ventilation and metabolic rates, rapid physical development, greater surface-to-volume ratios, and immature organ systems [20]. Young children cannot protect themselves from PYRs insecticides in households of low-income families which are not aware of health risks and toxic effects of PYR insecticide exposure. Parents should be knowledgeable and protect their children from contamination with PYR insecticides in their homes.

The impact of children’s behavior on exposure to environmental household contaminants has been described through studies of pesticides exposure. These studies show that hand-to-mouth activities and hygiene practices were associated with higher blood pesticides levels, on the hands, and in the diet. Pesticide exposure was also associated with object to mouth behavior, contact with upholstered furniture, and contact with bottles [4]. To date, few studies have investigated hand wipe sample concentrations of PYR insecticide exposure in children 2 to 3 years old in households where PYR mosquito coils and sprays are used every day. This study aims to identify associations between 3-PBA metabolites, hand wipe sample concentrations, and different exposure factors in children living in urban Bangkok, Thailand.

## Methods

### Study Site and Population

A cross-sectional study was conducted with 80 children aged 2–3 years old from Bangkok, Thailand. This study included children who were living in households which used PYR insecticide products daily for the control of insects, specifically mosquitos. We focused on children in low-income families because a PYR insecticide product is used daily and parents lacked the awareness or knowledge of how to protect their household from health risks. One parent per child was asked to complete questionnaires concerning demographic information and their children’s personal exposure. The children provided urine samples in their homes for subsequent 3-PBA metabolite determination and provided hand wipe samples. This study was approved by the Ethics Review Committee for Research Involving Human Research Subjects, Health Science Group, Chulalongkorn University, Thailand (COA No. 290/2561).

### Questionnaires

Parents provided key information concerning the children’s demographic characteristics, including age, and gender. Parents also completed a second questionnaire about themselves, including education level, occupational status and provided information on the care giver of the children. Other information collected included exposure characteristics in the home and children’s personal exposure.

### Urine and hand wipes collection

Children provided morning urine samples during our visits to their households. Urine samples were collected in standard polypropylene specimen containers; each child provided a 50-mL sample, and these were all stored at –20°C until later analysis. Wipe samples were collected from the hands of the children then frozen at –18°C to ensure preservation of the compounds including cypermethrin.

### Pyrethroids metabolite analysis

The urine samples were thawed to analyze the PYR metabolite 3-PBA. We selected this metabolite because it is formed by oxidation of the hydrolytic product of many PYR insecticides—such as cypermethrin, d-allethrin, imiprothrin, prallethrin, d-tetramethrin, metofluthrin, and esbiothrin—commonly found in mosquito coils and spray products. Furthermore, 3-PBA had been confirmed as among the most important metabolite biomarkers of PYR insecticide exposure in human populations.

To extract the PYR metabolite, we modified the procedures described by several authors [6,7,8]. Briefly, a 20-ml urine sample was transferred to a 50-ml glass vial with a screw top. For hydrolysis, 2 ml of hydrochloric acid (37%) was added and heated for 45 min at 70°C in a water bath. Samples were then cooled to room temperature. The urine samples were extracted twice, to which 8 ml of n-hexane was added by shaking for 10 minutes. These samples were centrifuged for 5 minutes at 2500 rpm and the supernatant removed and combined in new a 50-ml glass vial with a screw top. Cleanup by 4 ml of 0.1 N NaOH was added to the supernatant; this was centrifuged again for 5 min at 2500 rpm. The supernatant was transferred to a 2-ml autosampler vial. Each child-specific urine sample was extracted two times, following the same protocol, to obtain a final mixture of supernatants together with a higher concentration. The extract was evaporated to dryness by heating at 60°C for 5 minutes with nitrogen flow. The residue was dissolved in 100 ml of toluene. The solution was transferred to micro-vials and sealed with vial caps. For derivatization, the vials were heated at 70°C for 20 minutes in a water bath; a 1-ul subsample was then analyzed by GC/MS. In this study an internal quality standard was not added within the urine samples to check that the urine sample contained 3-PBA only, as it was known that the children were directly exposed to PYR insecticide from mosquito coils and sprays, which their parents used daily. The analysis of each urine sample was performed twice.

3-PBA metabolite detection and identification were achieved through a sensitive and selective capillary gas chromatographic procedure coupled to mass spectrometric detection (GC/MS), using conditions from Colume and Schettgen [6,8]. Briefly, the following GC/MS conditions were used: an operating temperature of the injector set to 280°C; chromatographic separation through a HP-5 capillary column (30 m, 0.25 mm I.D., 0.25-µm film thickness) purchased from Hewlett- Packard (Waldbronn, Germany); helium 5.0 as the carrier gas at a constant flow of 2.2 ml/min; an initial column temperature of 90°C held for 1 minute, then raised at a rate of 25°C/min to 120°C; then raised at a rate of 2.1°C/min to 240°C, and held at this temperature for 1.5 min; and finally raised at 25°C/min to 310°C, where it remained for 7 min. An injection volume of 1 ml was used for the retention times of the derivatized analytes. For each of the analytes, quantitative analysis of the PYR metabolites and selected ion monitoring (SIM) was used, with two or three fragment ions scanned (3-PBA M/e: 121, 214, 215).

### Hand wipe sample analysis

Hand wipes were placed in test tubes to which 25 mL of acetonitrile was added. After the sample was shaken for 10 min, the solvent was removed from the test tube. The extracted sample was evaporated under 60°C, using nitrogen gas, to almost dryness. A volume of 1 mL of acetonitrile containing 0.1% acetic acid v/v was then added and the sample was transferred to a 1-mL Eppendorf tube. The sample was vortex mixed for 1 min, and then centrifuged at 6,000 rpm for 2 min. The sample was then transferred to a 1.5-mL vial for cypermethrin analysis using gas chromatography (GC).

Cypermethrin detection and identification were achieved through a sensitive and selective capillary gas chromatographic procedure coupled to mass spectrometric detection (GC/MS), using conditions modified following 3-PBA metabolite detection.

### Statistical analysis

Data was presented as mean ± standard deviation. Categorical variables were reported as frequencies and percentages. The Kolmogorov-Smirnov test indicated the data distributions were non-normal. We used Spearman correlations to test the association between 3-PBA metabolites and hand wipe samples. We used binary logistic regression to test the association between 3-PBA metabolites and exposure factors including children’s information, children’s personal exposure and exposure characteristics, home characteristics, and dietary behavior. Association between hand wipe sample concentrations and exposure factors were also tested. A bivariate analysis of each variable was done first, and then variables with p-value < 0.25 were included in the multivariate analysis. Statistical analysis was performed using the Statistical Package for the Social Sciences Program (SPSS), version 22.

## Results

### Demographics

Children were 2–3 years old (n = 80), average 30 months, with slightly more 52 (65%) females than 28 (35%) males, and an average weight of 14.2 kg. A minority of the parents were illiterate (10%) while those who graduated primary school were a majority (90%). Most of the children had a parent who was unemployed (75%), with employed being (25%). Children who lived with the mother was 37.5% and 62.5% lived with another caregiver (Table 1).

**Table 1:** Characteristics and factors exposure in children of 2–3 years from Bangkok households (N = 80).

Characteristics/factors exposure	*n* (%) or (median ± SD)

Children and parent information	
Aged (months)	36.00 ± 11.15
Weight (kg)	13.6 ± 4.7
Gender	
Male	28 (35)
Female	52 (65)
Parents education level	
Illiterate	8 (10.0)
≥Primary school	72 (90.0)
Occupational status	
No Work	60 (75.0)
Work	20 (25.0)
Care giver of children	
Others	50 (62.5)
Mother	30 (37.5)
**Exposure characteristics**	

Type of PYR insecticide use	
Others insecticide	13 (16.3)
Coil insecticide	67 (83.8)
Frequency of PYR insecticide use in household (times/month)	
Some time	17 (21.3)
Every day	63 (78.8)
Storage PYR insecticide products	
Outside household	13 (16.2)
Inside household	67 (83.8)
Frequency of floor-cleaning	
Never	28 (35.0)
Once a week	52 (65.0)
Type floor cleaning	
Non-wet broom	28 (35.0)
Wet broom	52 (65.0)
**Children’s personal exposure**	

Children play of the floor in household	
No	
Yes	
Children play soil during the in household	
No	
Yes	
Children walk bare feet inside household	
No	18 (22.5)
Yes	62 (77.5)
Children wash their hands/feet during the day	
No	33 (41.3)
Yes	47 (58.8)
Children take a shower during the day	
1–2 times	38 (47.5)
>2 times	42 (52.5)
Children put hand-to-mouth	
No	12 (15.0)
Yes	67 (83.8)
Children put object-to-mouth	
No	14 (17.5)
Yes	66 (82.5)
Children put finger-to-mouth	
No	18 (22.5)
Yes	62 (77.5)
Eating on the floor	
No	14 (17.5)
Yes	66 (82.5)
Washing fruits	
No	28 (35)
Yes	52 (65)
**Home characteristics**	

Type of household	
Non permanency	28 (35)
Permanency	52 (65)
Main type floor	
Non cement floor	28 (35)
Cement floor	52 (65)

### Exposure characteristics

According to the information provided by the parents (Table 1), most households (83.8%) used the coil insecticide. The majority used insecticides daily (78.8%), and just a small proportion using insecticides occasionally (21.3%). Almost all households had their floors cleaned one time/week (65.0%), the majority using a wet broom (65.0%).

### Children’s personal exposure

Data as provided by the parents revealed that most of children play on the floor (78.8%), while fewer play on soil (35.0%). A majority walk bare foot inside the household (77.5%), wash hands and feet during the day (58.8%), take a shower during the day (52.5%), put hands and objects to the mouth and eat on the floor (82.5%), put fingers to the mouth (77.5%) and wash fruits before eating (65.0%).

### Home characteristics

According to the home characteristics provided by the parents (Table 1), most household types were permanent (65.0%) and the main floor type was cement (65.0%).

### The 3-PBA metabolite and hand wipe concentrations in children of 2–3 years old (n = 80) from Bangkok households

Concentrations of the 3-PBA metabolite and hand wipe concentrations are shown in (Table 2). Their detection was >90% in the children’s urine samples and hand wipe samples. 3-PBA metabolite concentrations measured in the range of 0.23 – 2.53 µg/mL with a median of 1.46 µg/mL and mean ± SD of 1.47 ± 0.77 µg/mL. In addition, cypermethrin insecticide showed a concentration of 0.016 ± 0.004 µg/mL.

**Table 2:** The 3-PBA metabolite and hand wipes concentration in children of 2–3 years (n = 80) from Bangkok households.

Biomarkers	Concentration			

	Median	Mean ± SD	Range	Detected (%)

3-PBA (µg/mL)^a^	1.460	1.470 ± 0.084	0.231–2.534	92
Hand wipes (µg/mL)^b^				
Cypermethrin	0.0171	0.016 ± 0.0049	0.021–0.005	92

^a^ Method detection limits (MDLs) = 0.01 µg/mL. ^b^ Method detection limits (MDLs) = 0.001 µg/mL.

The 3-PBA metabolite was positively correlated with hand wipe concentrations represented by cypermethrin (r_s_ = 0.226, p-value < 0.05) (Table 3). Moreover, the increase of 3-PBA metabolite was significantly associated with increased hand wipes sample concentrations (including cypermethrin).

**Table 3:** The correlation between 3-PBA metabolite and hand wipes sample of 2–3-year-old children (n = 80) from Bangkok households.

Urine sample	Spearman’s coefficient (r_s_)	

	3-PBA	Cypermethrin

3-PBA (µg/mL)	1.000	
Hand wipes (µg/mL)		
Cypermethrin	0.226*	1.000

* Correlation coefficient significant at p < 0.05.

The binary logistic regression test, represented by multivariate analysis, resulted in an association between exposure factors and 3-PBA metabolite concentration (Table 4). Gender presented a significant association with 3-PBA metabolites (p-value = 0.035). Females showed a 0.326-fold increase of 3-PBA metabolites more than males, but only presented a low risk exposure (OR = 0.326, 95% CI 0.115–0.926). This may be due to females spending more time within the household. Additionally, bare feet inside of the household presented a significant association with 3-PBA metabolites (p-value < 0.01), and if the children spent time on the floor with bare feet everyday there was an increase of 7.072-fold of 3-PBA metabolites compared to being non-bare feet inside the household, which presented a high risk exposure (OR = 7.072, 95% CI 1.707–29.291). Therefore, gender and bare feet inside household presented risk odds higher than other factors. In addition, age was not significantly associated with 3-PBA metabolites (p-value > 0.05).

**Table 4:** Multivariate analysis of factor’s exposure with 3-PBA metabolites concentration on urine children (n = 80) from Bangkok households.

Multi-factors exposure	B	S.E.	Wald	p-Value	OR (95% CI)

Aged (months)	–0.008	0.023	0.124	0.725	0.992 (0.949–1.037)
Gender^a^	–1.120	0.532	4.428	0.035*	0.326 (0.115–0.926)
Walk bare feet inside household^b^	1.956	1.418	3.983	0.007*	7.072 (1.707–29.291)

3-PBA metabolite concentration in urine (Low = ≤ 1.46 µg/mL, High = ≥ 1.46 µg/mL). B, regression coefficient; S.E., standard error; OR, odds ratio; CI, confidence interval. Reference category: first. ^a^ 0 = male, 1 = female. ^b^ 0 = no, 1 = yes. *Significant at p < 0.05.

A multivariate analysis of exposure factors with hand wipe sample concentration (cypermethrin) was run (Table 5). All exposure factors were not significantly associated with wipe sample concentrations (cypermethrin). However, our results showed gender did not present a significant association with cypermethrin (p-value > 0.05). Females showed a 2.058-fold increase of 3-PBA metabolites over males, but presented an exposure of (OR = 2.058, 95%CI 0.696–6.087). In addition, walking bare foot inside the household, playing on the floor and playing in soil did not present a significant association with cypermethrin (p-value > 0.05), but presented in high risk of exposure (OR = 2.282, 95% CI 0.396–13.166; OR = 2.143, 95% CI 0.357–12.851 and OR = 4.189, 95% CI 0.501–35.015, respectively).

**Table 5:** Multivariate analysis of factor’s exposure with hand wipe sample (Cypermethrin) in children (n = 80) from Bangkok households.

Multi-factors exposure	B	S.E.	Wald	p-Value	OR (95% CI)

Aged (months)	0.019	0.023	0.677	0.411	1.019 (0.974–1.066)
Gender^a^	0.722	0.553	1.700	0.192	2.058 (0.696–6.087)
Walk bare feet inside househol^d^b	0.825	0.894	0.852	0.356	2.282 (0.396–13.166)
Play of the floor in househol^d^b	0.762	0.914	0.696	0.404	2.143 (0.357–12.851)
Play soil during the in househol^d^b	1.432	1.083	1.748	0.186	4.189 (0.501–35.015)

3-PBA metabolite concentration in urine (Low = ≤0.0171 µg/mL, High = ≥0.0171 µg/mL). B, regression coefficient; S.E., standard error; OR, odds ratio; CI, confidence interval. Reference category: first. ^a^ 0 = male, 1 = female. ^b^ 0 = no, 1 = yes. * Significant at p < 0.05.

## Discussion

This study focused on young children (2–3 years old) in Bangkok, Thailand, similar to a previous study related to insecticides imported into Thailand [3]. Our study focused on children in low-income families because the parents used PYR insecticide products daily and generally lacked the awareness or knowledge of how to protect a child against exposure, these criteria agrees with Quiras-Alcal and Kunno [9,18].

Concentrations of 3-PBA metabolite were measured at 1.46 µg/mL. This situation is directly dangerous to young children because they spend a significant amount of time inside the household all day and can be exposed via dermal exposure or inhalation. Moreover, children can be potentially exposed to PYR components from food and other products that contain of PYR at home [5,10]. In addition, our research found 3-PBA metabolite concentrations higher than past studies [10,11,12]. These results should lead to certain recommendations in order to reduce the risk of 3-PBA metabolite exposure. These recommendations include promoting health education and risk management. This population, children from Bangkok living in urban households, was found to have more 3-PBA metabolites in their urine samples than children from agricultural regions. One suggestion of the origin of the 3-PBA metabolites is from exposure to permethrin and cypermethrin and this finding agrees with [13]. This finding was supplemented from hand wipe concentrations in children aged 2–3 years old. The mean cypermethrin concentration was 0.016 µg/mL. Potential factors impacting this result could be contact behavior such as putting objects in the mouth, putting hands to the mouth etc., or the frequency of PYR use. This study, like a previous a study related to hand wipe samples which measured 0.096–0.503 µg/hand, confirmed that children’s hands, feet, and toys may accumulate pesticide residues [3].

A correlation was found between 3-PBA metabolites and hand wipe sample concentrations. The 3-PBA metabolite concentration in children’s urine samples presented a significant relationship to hand wipe sample concentrations (r_s_ = 0.226–0.274, p-value < 0.05). Moreover, the increase of 3-PBA metabolite was significantly associated with an increase in hand wipe sample concentrations (including cypermethrin). This study finding agrees with Siriwat [3]. In addition, exposure estimation suggests that hand-to-mouth contact represents another important pathway along with dust ingestion and that children are subject to higher pesticide exposure than adults [14,15]. Pesticide exposures are highly prevalent, and data provided herein further substantiates hand-to-mouth contact and dermal absorption as important pathways of pesticide exposure, especially for youth [16,17,18].

The binary logistic regression test, represented by multivariate analysis, presented an association between exposure factors with 3-PBA metabolite concentration. While, gender and bare feet inside households were significantly associated with increased 3-PBA metabolite concentrations. In addition, exposure factors were not significantly associated with wipe sample concentration (cypermethrin) but could possibly indicate high risk exposure. Finally, young children are not able to change their low-income status or protect themselves from PYR use in their household and the parents tend not to be aware of health risks and toxic effects of PYR insecticide exposure. Therefore, we suggest there should be education, increased awareness, and management to reduce the risk from long-term PYR insecticide exposure of children living in households.

## Conclusion

This study focused on PYR exposure to young children in households via dermal routes, mainly hands wipe samples. In this study 3-PBA metabolite concentrations were measured at 1.46 µg/mL and the finding was supplemented from hand wipe concentrations in children aged 2–3 years old. The mean cypermethrin concentration was 0.016 µg/mL. Potential factors impacting this result could be contact behavior such as putting objects in the mouth, putting hands to the mouth etc., or the frequency of PYR use. The study results suggest hands might be the main PYR exposure pathway in young children. Using coil insecticides daily may serve as the one factor for PYR exposure to children who walk with bare feet inside households and have object-to-mouth behavior. These findings should be verified in a future investigation with a longitudinal design and a larger study population. The recommendations of this study are that parents in Bangkok be given education, increased awareness, and management support to reduce their children’s risk from long-term PYR insecticide exposure in urban households.
